# Molecular characterisation of viral pathogens associated with respiratory and gastrointestinal infections in dogs in Türkiye – preliminary study

**DOI:** 10.2478/jvetres-2026-0003

**Published:** 2026-02-04

**Authors:** Hatice Pelin Aslim, Rüveyde Gülbahçe, Irmak Dik, Hasan Sercan Palanci, Oya Bulut

**Affiliations:** Selçuk University, Faculty of Veterinary Medicine, Department of Virology, Konya 42130, Türkiye; Dokuz Eylul University, Kiraz Vocational School, Izmir 35890, Türkiye; Dokuz Eylul University, Faculty of Medicine, Department of Medical Microbiology, İzmir 35330, Türkiye

**Keywords:** canine adenovirus, canine parvovirus, canine distemper virus, canine herpesvirus

## Abstract

**Introduction:**

Canine viral infections cause significant morbidity and mortality in dogs worldwide. This study aimed to investigate the presence of canine adenovirus (CAdV), canine parvovirus (CPV), canine distemper virus (CDV) and canine herpesvirus (CHV) at the molecular level.

**Material and Methods:**

A total of 68 paired nasal secretion and blood samples were obtained from 34 dogs, and 93 faecal samples were collected, each from a single dog. All sampled animals showed clinical signs of respiratory or gastrointestinal disorders. They came from five different provinces of Türkiye. The samples were tested by PCR and selected strains were sequenced.

**Results:**

While no CAdV was detected in the PCR analyses, CPV gene amplification was achieved in 60.2% (56/93) of the DNA extracted from faecal samples, CDV genes were amplified in 11.8% (4/34) of the genetic material extracted from nasal swabs, and CHV genes were amplified in 14.7% (5/34). One nasal swab sample showed a co-infection with CDV and CHV, but the corresponding blood sample did not. Phylogenetic analyses of the viral strains were conducted; among CPV strains, CPV-2b and CPV-2c variants were identified and found to share high genetic similarity with strains of Asian and African origin. The CDV strains were closely related to European strains, while the CHV strains exhibited genetic diversity and matched strains isolated worldwide. No statistically significant association was found between viral infections and the sex or age of the animals.

**Conclusion:**

These findings provide insight into the molecular epidemiology of viral infections in dogs in Türkiye and reveal that local strains are phylogenetically closely related to globally circulating strains.

## Introduction

The number of companion dogs is continuously increasing in various countries around the world. Accordingly, viral gastrointestinal and respiratory infections such as canine adenovirus (CAdV), canine parvovirus (CPV), canine distemper virus (CDV), and canine herpesvirus (CHV) continue to pose significant health risks to dogs, with their importance increasing in parallel with the rising number of companion animals worldwide, potentially affecting a growing dog population (2, 15). Clinical samples frequently show co-infections involving two or more of these viruses with other gastrointestinal or respiratory pathogens, and these exacerbate disease severity through more marked clinical manifestations and pathological lesions (23, 29). Therefore, accurate and reliable detection and differentiation of these pathogens are of vital importance for the diagnosis and treatment of canine viral diseases (26).

Canine adenovirus, particularly type 1 (CAdV-1), causes infectious canine hepatitis, a serious and potentially fatal liver disease in dogs, while type 2 (CAdV-2) is commonly associated with infectious tracheobronchitis and enteritis. Canine adenovirus belongs to the *Mastadenovirus* genus of the *Adenoviridae* family and features a linear, double-stranded DNA genome approximately 20–30 kb in length (35). Canine parvoviral disease is a highly contagious and often fatal infection caused by canine parvovirus type 2 (CPV-2), typically presenting with severe vomiting and haemorrhagic diarrhoea. The type-2 virus, first described in the late 1970s following mutation of feline panleukopenia virus, shares over 98% genetic similarity with its progenitor. New antigenic variants known as CPV-2a, 2b and 2c have since emerged and replaced the original CPV-2 strain globally (30). The variants and the original are small, non-enveloped viruses of approximately 25 nm, classified under the *Protoparvovirus* genus of the *Parvoviridae* family, with a linear, single-stranded DNA genome about 5 kb in length (14, 22).

Canine distemper virus (CDV) is a highly contagious agent causing canine distemper, characterised by fever, respiratory infections, leukopenia and neurological symptoms (34). It is a negative-sense, non-segmented, single-stranded RNA virus with 15,960 nucleotides, enveloped by a lipoprotein membrane, and belongs to the *Morbillivirus* genus in the *Paramyxoviridae* family (12). Canine herpesvirus type 1 (CHV-1) infection is one of the most widespread diseases in both domestic and wild canines worldwide. It typically causes systemic and often fatal illness in puppies under three weeks of age, and results in respiratory, urinary, reproductive, ocular and neurological disorders in adult dogs. The aetiological agent is a DNA virus belonging to the *Varicellovirus* genus of the *Alphaherpesvirinae* subfamily of the *Herpesviridae* family (32).

The prevalence and distribution of co-infections involving CAdV, CPV, CDV and CHV in Türkiye remain unclear. Therefore, the aim of the study was to investigate the presence of CAdV, CPV, CDV and CHV *via* PCR in faecal samples, respiratory secretions and blood samples from dogs showing respiratory or gastrointestinal symptoms in the Muğla, Kahramanmaraş, Afyon, Konya and Denizli provinces of Türkiye. Target genes with high diagnostic sensitivity and the potential to reflect genetic variation were selected: early gene 3 (E3) for CAdV, viral capsid protein 2 (VP2) for CPV, haemagglutinin (H) for CDV and thymidine kinase (TK) for CHV. Additionally, genetic material from selected positive samples was characterised molecularly to determine the Turkish viruses’ phylogenetic relationships with global strains.

## Material and Methods

### Sample collection and preparation for analysis

Samples were collected from 127 Turkish dogs showing clinical signs related to either respiratory or gastrointestinal system infections. Faecal samples from 18 animals and nasal swab and blood samples from 5 animals were collected in the province of Muğla, faecal samples from 6 animals and nasal swab and blood samples from 4 animals were provided from Kahramanmaraş, 10 dogs and 7 dogs were sampled in this scheme in Afyon, Konya province was the origin of 28 faecal samples and 18 nasal swab and blood samples, and from Denizli only faecal samples were taken from 31 animals. A total of 93 faecal samples were collected from dogs with gastrointestinal symptoms, and 34 pairs of a nasal swab and a blood sample (*i.e*. 68 samples in total) from dogs showing respiratory symptoms. All specimens were transported under cold chain conditions to the Virology Laboratory of the Faculty of Veterinary Medicine at Selçuk University.

Blood samples collected in EDTA-containing tubes were centrifuged at 3,000 rpm for 5 min. Following centrifugation, the leukocyte layer was aseptically separated and transferred into 1.8 mL centrifuge tubes.

Similarly, faecal samples were diluted at a ratio of 1:10 in PBS containing 1% penicillin (1,000–2,000 IU/mL) and centrifuged at 4,000 rpm for 15 min. The obtained supernatants were filtered through 0.22-μm-pore cellulose acetate filters, aliquoted into 2-mL Eppendorf tubes in 1 mL volumes, and used for nucleic acid extraction. Respiratory secretion samples were withdrawn from swab tubes containing 1 mL of PBS using a syringe, filtered through 0.22-μm-pore cellulose acetate filters, and directly used for nucleic acid extraction. Viral DNA and RNA extractions were performed using the QIAamp DNA Mini Kit and QIAamp Viral RNA Mini Kit (cat. Nos. 51306 and 52904; Qiagen, Hilden, Germany), respectively, according to the manufacturer’s instructions.

### PCR analysis and gel visualisation

In this study, PCR analyses were performed for five commonly encountered viral pathogens associated with respiratory and gastrointestinal diseases in dogs, specifically CAdV-1, CAdV-2, CPV, CDV and CHV-1. A conventional PCR was used for CAdV-1/2 and CPV detection, a reverse-transcription PCR (RT-PCR) was used for CDV and a nested PCR was employed to detect CHV-1 nucleic acids. Commercially available kits (cat. No. G597; MegaFi Pro One-Step RT-PCR Kit, Applied Biological Materials, Richmond, BC, Canada and cat No. 04-12-00125; FIREPol Master Mix, Solis Biodyne, Tartu, Estonia) were used in the reactions, which were all carried out in a T100 thermocycler (Bio-Rad, Hercules, CA, USA). All PCR products were subjected to electrophoresis in 2% agarose gels at 100 V and 80 mA for 50 min. The bands were then visualised using a gel documentation system.

### Detection of CAdV-1/2

The presence of CAdV-1/2 was investigated using primers specific to the E3 gene region of the virus. Genetic material extracted from all 161 samples (93 faecal, 34 nasal secretion and 34 blood) was assayed. The primers from the study of Hu *et al*. (17) were used, which yield a 509-bp product for CAdV-1 and a 1,031-bp product for CAdV-2. The forward primer sequence was 5′-CGCGCTGAACATTA CTACCTTGTC-3′ and the reverse primer sequence was 5′-CCTAGAGCACTTCGTGTCCGCTT-3′.

The PCR reaction mixture was prepared to a total volume of 40 μL, containing 8 μL of 5× Master Mix, 1 μL of forward primer (10 pmol), 1 μL of reverse primer (10 pmol), 26 μL of nuclease-free water and 4 μL of DNA template. The thermal cycling profile consisted of initial denaturation at 95°C for 7 min; 30 cycles of denaturation at 95°C for 30 s, annealing at 58°C for 60 s and extension at 72°C for 90 s; and a final extension step at 72°C for 7 min.

### Detection of CPV

Only the genetic material extracted from faecal samples was analysed by PCR to investigate the presence of CPV nucleic acid. For this purpose, primer sequences targeting a specific 630-bp gene region and partially amplifying the capsid protein-coding VP2 gene of the virus were selected, as used in the study by Buonavoglia *et al*. (6). The forward primer used was 5 ′-CAGGTGATGAATTTGCTACA-3′ and the reverse primer was 5′-CATTTGGATAAACTGGTGGT-3′.

The PCR reaction mixture was prepared with a total volume of 40 μL: 8 μL of 5× Master Mix, 1 μL of forward primer (10 pmol), 1 μL of reverse primer (10 pmol), 26 μL of nuclease-free water, and 4 μL of DNA template. The PCR cycling conditions were an initial denaturation at 95°C for 7 min; 30 cycles of denaturation at 95°C for 30 s, annealing at 55°C for 50 s and extension at 72°C for 60 s; and a final extension step at 72°C for 7 min.

### Detection of CDV

To investigate the presence of CDV nucleic acid, all 161 samples were used. A forward primer with the 5′-TGCTCTCCTACCAAGACAAGG-3′ sequence and a reverse primer with the 5′-TCAAGGTTTTGAACGGTTACATG-3′ sequence were selected, which can amplify the entire haemagglutinin (H) gene region of the virus as described by Bhatt *et al*. (4). Using these primers, a 1,823-bp PCR product was obtained.

The reaction mixture was prepared with a total volume of 50 μL: 25 μL of 2× buffer, 4 μL of enzyme mix, 2.5 μL of forward primer (10 pmol), 2.5 μL of reverse primer (10 pmol), 11 μL of nuclease-free water and 5 μL of RNA template. The PCR cycle started with a reverse transcription step at 60°C for 15 min; continued with an initial denaturation at 95°C for 5 min and 32 cycles of denaturation at 95°C for 15 s, annealing at 54°C for 50 s and extension at 72°C for 70 s; and concluded with an extension step at 72°C for 5 min.

### Detection of CHV-1

This virus was investigated only in the nasal swabs and blood samples. A conventional nested PCR was applied for the molecular detection of CHV-1. The PCR primer sets used in the study were based on those used by Bottinelli *et al*. (5), targeting the *TK* gene.

In the first round of PCR, the 5′-TGCCGCTTT TATATAGATG-3′ forward primer and 5′-AAGCGTTGT AAAGTTCGT-3′ reverse primer were used to amplify a 493-bp product. In the second round, the 5′-CGTGGTGAATTAAGCCTAA-3′ forward primer and 5′-ATGCTATTGGGGTGTCTATC-3′ reverse primer were used to amplify a 170-bp product. The template for the second PCR was the PCR product obtained from the first round.

Both PCR reactions were prepared with a total volume of 40 μL. In the first step, 8 μL of 5× Master Mix, 1 μL of forward primer, 1 μL of reverse primer, 26 μL of nuclease-free water and 4 μL of DNA template were used. In the second step, 8 μL of 5× Master Mix, 1 μL of forward primer, 1 μL of reverse primer, 28 μL of nuclease-free water and 2 μL of first-round PCR product as template DNA were used.

For the first PCR step, the cycling conditions included an initial denaturation at 95°C for 5 min; 30 cycles of denaturation at 95°C for 30 s, annealing at 55°C for 50 s and extension at 72°C for 50 s; and a final extension at 72°C for 7 min. In the second step, the conditions included an initial denaturation at 95°C for 5 min; 28 cycles of denaturation at 95°C for 30 s, annealing at 56°C for 50 s and extension at 72°C for 50 s; and a final extension at 72°C for 7 min.

### Sequence analysis

Sequencing of the DNA of four PCR products obtained from CPV (two from female and two from male dogs), four products obtained from CDV (two from male and two from female dogs) and five from CHV (three from male and two from female dogs) was conducted through outsourced services provided by the Medsantek company (Istanbul, Türkiye). The obtained sequences were compared with other sequences available in the GenBank database and aligned using the ClustalW method in BioEdit software v.7.0.5.3 (13). For the construction of the phylogenetic tree, MEGA software v. 4.1 (19) was used, and the tree was created using the neighbour-joining method. Bootstrap values were calculated with 1,000 replicates.

### Statistical analysis

The data obtained in the study were statistically analysed using SPSS 25.0 (SPSS, Chicago, IL, USA). To determine whether there was a significant difference in viral presence between faeces and swab samples, data obtained for CDV were subjected to the chi-squared test. To assess whether there were significant differences in pathogen presence correlated with sex, data were analysed using the Mann–Whitney U test. To assess whether there were significant differences by dog age in sample positivity for CPV, CDV and CHV, prevalence results were analysed using the Kruskal–Wallis test.

## Results

**Fig. 1.**
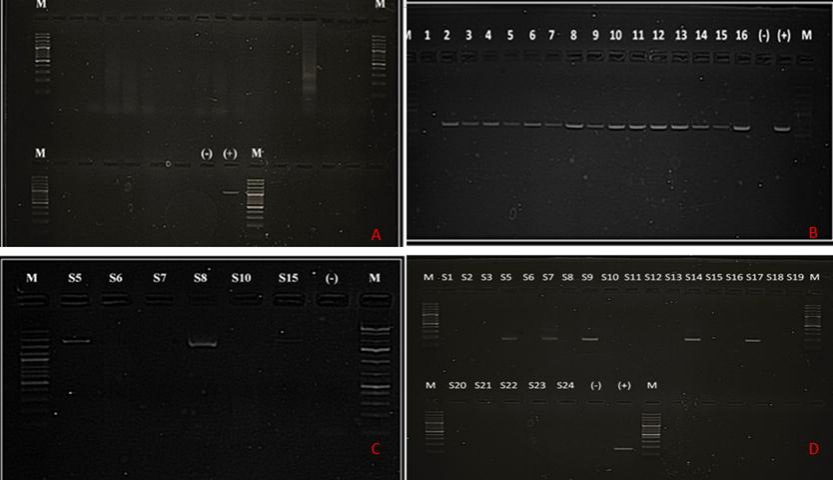
(A) Gel image of PCR results with CAdV-1/2-specific primer sets. All 24 samples of the full assortment were negative for CAdV-1/2. M – 100 bp marker; (-) – negative control; (+) – CAdV-2 positive control showing a band at 1,031 bp. (B) Gel image of PCR results with CPV-specific primer sets. Fifteen out of sixteen faecal samples were positive for CPV. M – 100 bp marker; (-) – negative control; (+) – CPV positive control showing a band at 630 bp. (C) Gel image of PCR results with CDV-specific primer sets. Three out of six nasal swab samples were positive for CDV. M – marker; (-) – negative control. (D) Gel images of second-round nested PCR results with CHV-1-specific primer sets. Five out of twenty-three nasal swab samples were positive for CHV-1. M – marker; (-) – negative control; (+) – CHV-1 positive control showing a band at 170 bp

No specific CAdV-1/2 genes could be amplified through PCR analysis of the genetic material extracted from samples of any type (Fig. 1A).

Specific CPV genes were amplified from 56 of the 93 faecal samples obtained from dogs. Specific CDV genes were amplified from nasal swab samples from four dogs showing respiratory infection symptoms, while none were from the blood samples from these animals. No CDV gene amplification was achieved from any of the 93 faecal samples obtained from animals with gastroenteritis. Specific CHV-1 genes were amplified from nasal swab samples from five dogs, while similarly, none were from the blood samples from these animals (Fig. 1). In nasal secretion sample number 5 (S5), co-infection with CDV and CHV-1 was detected.

### Sequence analysis results

A phylogenetic tree was constructed to reveal the characteristics of the 56 CPV-positive samples (4 of which were selected as representative), the 4 CDV-positive samples (2 of which were selected), and all 5 CHV-1-positive strains. Phylogenetic analyses of the *VP2* (CPV), *H* (CDV) and *TK* (CHV) gene sequences revealed high similarity among the strains and low evolutionary distances.

**Fig. 2.**
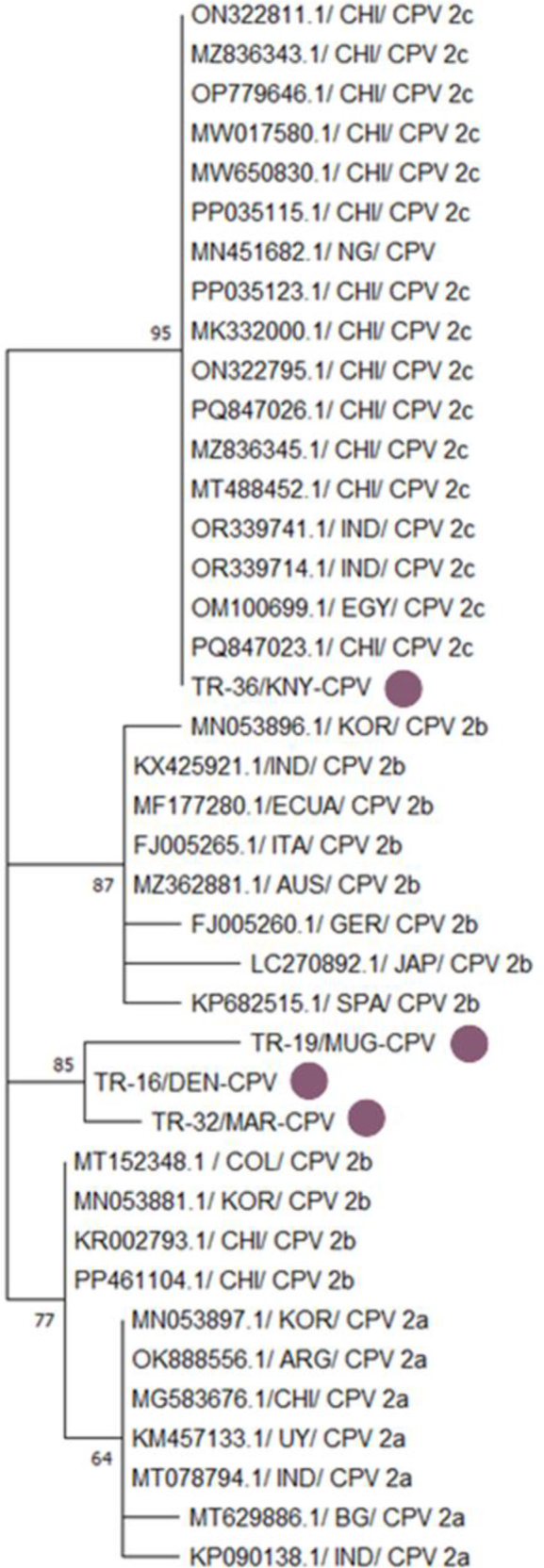
Phylogenetic tree based on the partial VP2 gene of canine parvovirustype 2 (CPV 2) constructed using the neighbour-joining method. Bootstrap analysis was performed with 1,000 repetitions. CHI – Chinese origin; NG – Nigerian origin; IND – Indian origin; EGY – Egyptian origin; KNY – origin in Konya province of Türkiye; KOR – South Korean origin; ECUA – Ecuadorian origin; ITA – Italian origin; AUS – Australian origin; GER – German origin; JAP – Japanese origin; SPA – Spanish origin; MUG – origin in Muğla province of Türkiye; DEN – origin in Denizli province of Türkiye; MAR – origin in Kahramanmaraş province of Türkiye; COL – Colombian origin; ARG – Argentinian origin; UY – Uruguayan origin; BG – Bangladeshi origin. Purple dots indicate isolates obtained in the present research. Numbers on tree branches are bootstrap support values

**Fig. 3.**
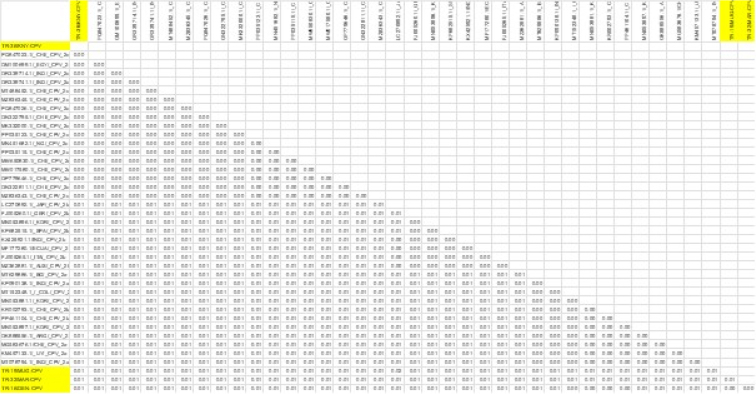
Results of genetic distance analysis of canine parvovirus type 2 (CPV-2) sequences. VP2-region sequences of CPV-2 isolates were obtained from the NCBI BLAST database

Four CPV strains isolated from different regions of Türkiye – TR-36/KNY-CPV, TR-19/MUG-CPV, TR-32/MAR-CPV and TR-16/DEN-CPV – were compared with reference CPV strains from around the world. In the phylogenetic tree, the TR-36/KNY-CPV strain branched as a CPV-2c variant and clustered within the same monophyletic group as CPV-2c strains from Asia (China and India) and Africa (Nigeria and Egypt). In contrast, the TR-19/MUG-CPV, TR-32/MAR-CPV and TR-16/DEN-CPV strains branched as CPV-2b variants and clustered within the same subclade. These three Turkish strains showed high similarity to CPV-2b strains reported from South Korea, China, Colombia and Ecuador, with a bootstrap value of 85 (Figs 2 and 3). This suggests that the CPV-2b variant is still circulating in Türkiye, and that local strains are phylogenetically related to international strains.

**Fig. 4.**
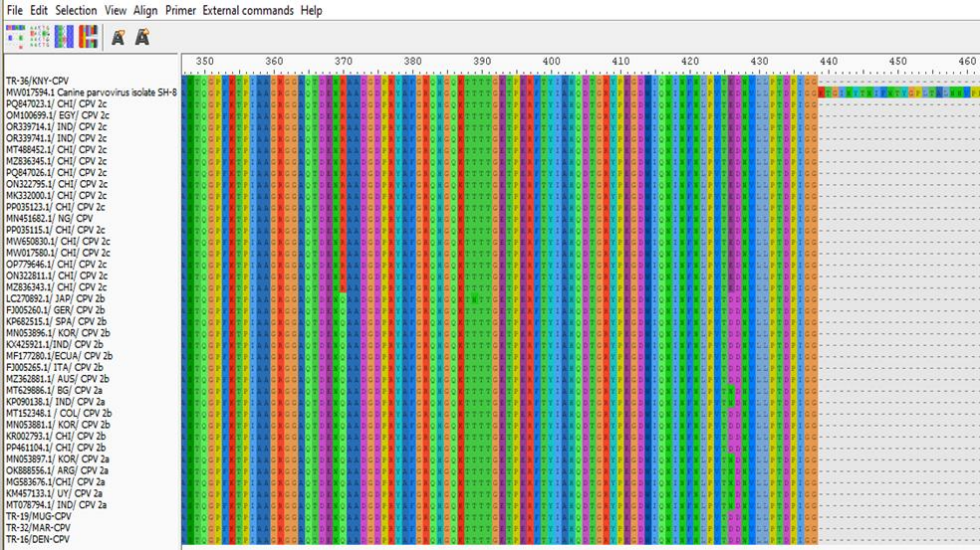
Canine parvovirus type 2 (CPV-2) amino acid changes in Turkish strains isolated in this study (at the top and bottom of the list) and strains logged in GenBank

Examination of the critical amino acid positions revealed that on the VP2 protein, which plays a significant role in determining host range, the TR-36/KNY-CPV strain carried the ala amino acid at position 297, consistent with the CPV-2c variant. Glycine at position 300, tyrosine at position 305 and asparagine at position 323 were conserved across all strains. At position 370, most strains (including TR-19/MUG-CPV, TR-16/DEN-CPV and TR-32/MAR-CPV) – had glutamine, while the TR-36/KNY-CPV strain had arginine, a substitution also observed in strains from other countries. At position 426, the TR-36/KNY-CPV strain carried glutamic acid, characteristic of the CPV-2c variant, whereas the CPV-2b strains – TR-19/MUG-CPV, TR-32/MAR-CPV and TR-16/DEN-CPV – had aspartic acid at the same position (Fig. 4).

**Fig. 5.**
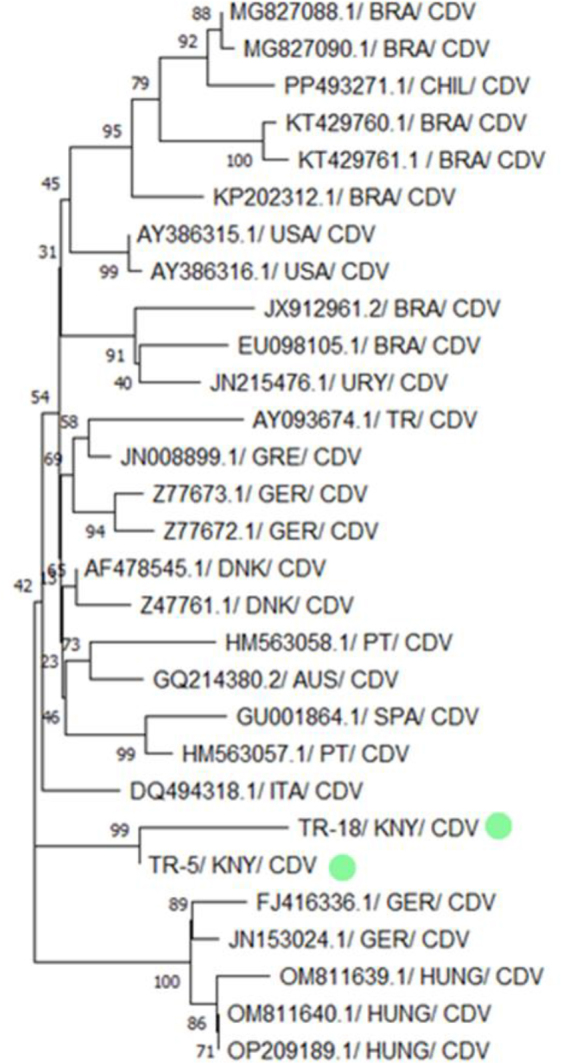
Phylogenetic tree based on the partial H gene of canine distemper virus (CDV) constructed using the neighbour-joining method. Bootstrap analysis was performed with 1,000 repetitions. BRA – Brazilian origin; CHIL – Chilean origin; USA – US American origin; URY – Uruguayan origin; TR – Turkish origin; GRE – Greek origin; GER – German origin; DNK – Danish origin; PT – Portuguese origin; AUS – Austrian origin; SPA – Spanish origin; ITA – Italian origin; KNY – origin in Konya province of Türkiye; HUNG – Hungarian origin. Green dots indicate isolates obtained in the present research. Numbers on tree branches are bootstrap support values

**Fig. 6.**
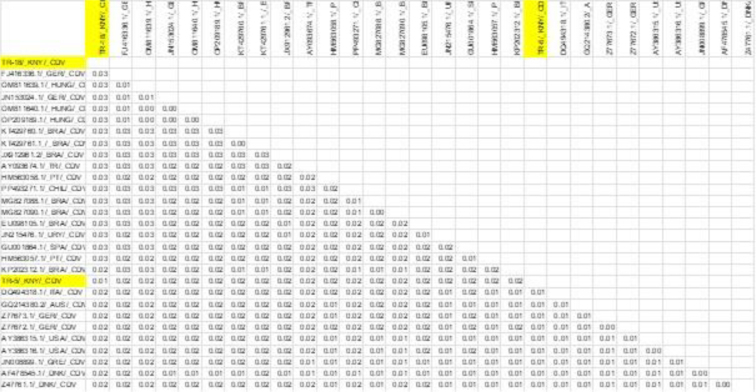
Results of genetic distance analysis of canine distemper virus (CDV) sequences. H-region sequences of CDV isolates were obtained from the NCBI BLAST database. Sequences obtained in the present research are highlighted in yellow

Two CDV strains isolated in this study – TR-18/KNY/CDV and TR-5/KNY/CDV – formed a close monophyletic group in the phylogenetic tree, particularly with strains from Hungary (OM811639.1, OM811640.1 and OP209189.1) and Germany (JQ153024.1 and FJ416336.1), evidenced by a high bootstrap value of 99. This finding was also supported by BLAST analysis, which revealed that the TR-18/KNY/CDV and TR-5/KNY/CDV strains shared ≥98% nucleotide similarity with these European strains (Figs 5 and 6).

**Fig. 7.**
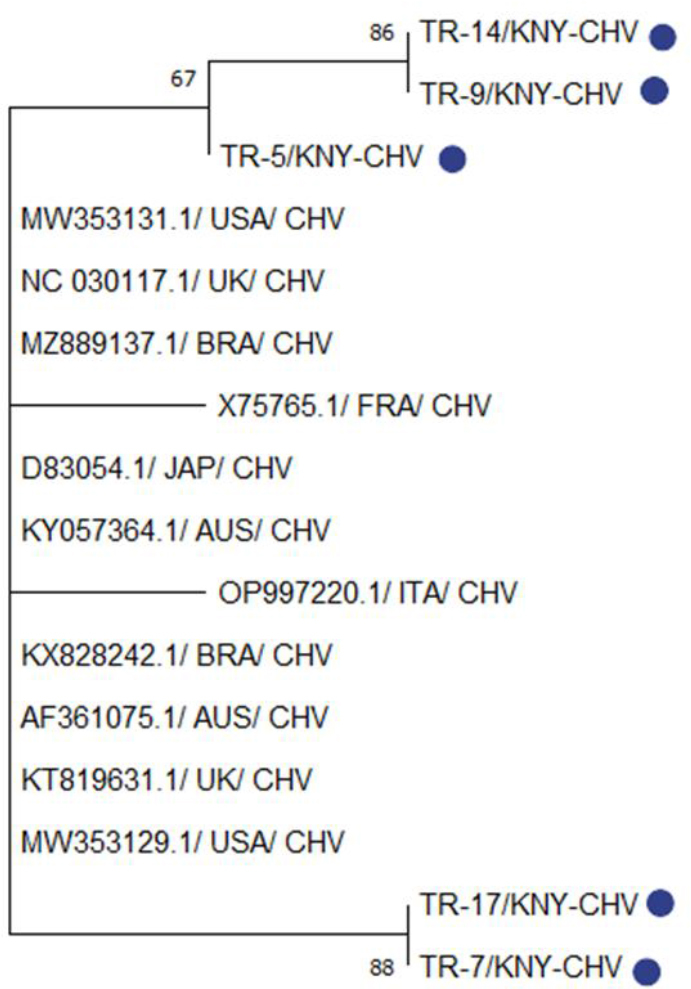
Phylogenetic tree based on the partial TK gene of canine herpesvirus (CHV) constructed using the neighbour-joining method. Bootstrap analysis was performed with 1,000 repetitions. KNY – origin in Konya province of Türkiye; USA – US American origin; UK – United Kingdom origin; BRA – Brazilian origin; FRA – French origin; JAP – Japanese origin; AUS – Australian origin; ITA – Italian origin. Blue dots indicate isolates obtained in the present research. Numbers on tree branches are bootstrap support values

**Fig. 8.**
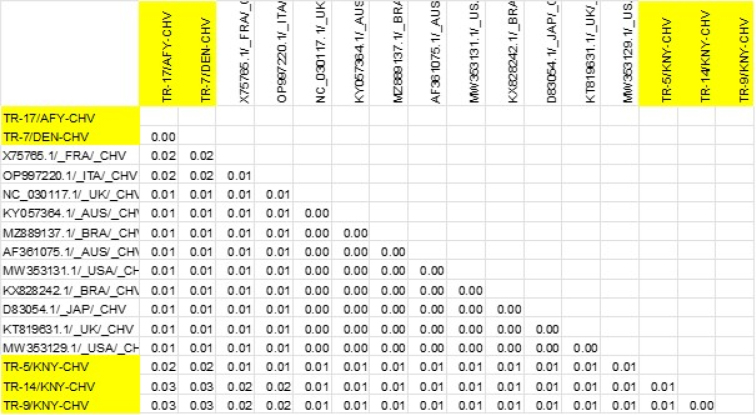
Results of genetic distance analysis of canine herpesvirus (CHV) sequences. TK-region sequences of CHV isolates were obtained from the NCBI BLAST database. Sequences obtained in the present research are highlighted in yellow

The five CHV strains isolated in this study – TR-5/KNY-CHV, TR-7/DEN-CHV, TR-9/KNY-CHV, TR-14/KNY-CHV and TR-17/AFY-CHV – were phylogenetically analysed together with CHV strains reported from different parts of the world. A clade was formed by TR-14/KNY-CHV and TR-9/KNY-CHV with strong bootstrap support of 86, and both strains appeared to share a more recent common ancestor with TR-5/KNY-CHV, suggesting that these three were genetically closely related and may have originated from a common source. Although they belonged to the same clade, these strains were phylogenetically distinct from CHV strains reported from the United States (MW353131.1), the United Kingdom (NC030117.1), Brazil (MZ889137.1) and France (X75765.1). In contrast, TR-17/AFY-CHV and TR-7/DEN-CHV were located on a separate branch of the phylogenetic tree and grouped together with strong bootstrap support of 88. These two strains appeared to be more closely related to CHV strains reported from the USA (MW353129.1), Australia (AF361075.1), the UK (KT819631.1) and Italy (OP997220.1) (Figs 7 and 8).

**Table 1.** Distribution of pathogen positivity in terms of sample type

Pathogen	Faeces				Swab				Total			
	Positive		Negative		Positive		Negative		Positive		Negative	
	N	%	n	%	n	%	n	%	n	%	n	%
Distemper virus	0	0	93	100	4	11.8	30	88.2	4	3.1	123	96.9
Statistical values: P-value < 0.05; χ^2^ = 11.297												
Parvovirus	56	60.2	37	39.8	No analysis performed				56	60.2	37	39.8
Herpesvirus	No analysis performed				5	14.7	29	85.3	5	14.7	29	85.3

## Discussion

In this study, the presence of CAdV, CPV and CDV viral agents was investigated in faecal samples collected from dogs showing clinical signs related to the digestive system. The proportion of CPV-positive males was 10% higher than that of females (31/56, 55% *vs* 22/56, 45%), but it did not constitute a statistically significant difference (Table 2), and this finding is consistent with the results reported by Franzo *et al*. (11). Similarly, no statistically significant relationship was found between CPV positivity and age (Table 3); this finding is consistent with the results reported by Yang *et al*. (31) in street dogs. The limited effect of individual factors such as sex and age on susceptibility to CPV infection likely reflects the influence of more complex interactions, such as between these factors and the widespread environmental presence of the virus, its high transmissibility and the individual dog’s immune status. Variables such as the presence of maternal antibodies and environmental stress factors may also prevent age alone from being a determining factor. This suggests a multifactorial basis for CPV infection.

**Table 2.** Distribution of pathogen positivity in terms of sex

Pathogen	Sex											
	Female				Male				Total			
	Positive		Negative		Positive		Negative		Positive		Negative	
	N	%	n	%	n	%	n	%	n	%	n	%
Distemper virus* (Swab analysis**)	2	1.6	60	47.2	2	1.6	63	49.6	4	3.1	123	96.9
Parvovirus* (Faecal analysis**)	25	26.9	23	24.7	31	33.3	14	15.1	56	60.2	37	39.8
Herpesvirus* (Swab analysis**)	3	8.8	11	32.4	2	5.9	18	52.9	5	14.7	29	85.3

* – no statistically significant differences were found in terms of sex in the analyses conducted for distemper virus, parvovirus or herpesvirus;

** – data from analysis groups with all-negative results were not included in the table

**Table 3.** Distribution of pathogen positivity in terms of age

Pathogen	Age															
	0–12 months				13–48 months				49 months and older				Total			
	Positive		Negative		Positive		Negative		Positive		Negative		Positive		Negative	
	n	%	n	%	n	%	n	%	n	%	n	%	n	%	n	%
Distemper virus* (Swab analysis**)	1	0.8	56	44.1	2	1.6	37	29.1	1	0.8	30	23.6	4	3.1	123	96.9
Parvovirus* (Faecal analysis**)	28	30.1	16	17.2	15	16.1	12	12.9	13	14.0	9	9.7	56	60.2	37	39.8
Herpesvirus* (Swab analysis**)	2	5.9	11	32.4	3	8.8	9	26.5	0	0	9	26.5	5	14.7	29	85.3

* – no significant differences were found in terms of age in the statistical analyses of distemper virus, parvovirus or herpesvirus;

** – data from analysis groups with all-negative results were not included in the table

When used for diagnostic purposes, PCR has ensured the accurate diagnosis of CPV infections with high sensitivity and specificity rates. Polymerase chain reaction also gave negative results for CAdV and CDV in all faecal samples. This known PCR reliability and the absence of CAdV and CDV in faecal samples strongly support the association of the observed gastrointestinal symptoms with CPV. In the 37 (39.8%) symptomatic animals in which CPV was not detected, it is thought that the symptoms may have been caused by other viral (*e.g*. canine coronavirus), bacterial (*e.g. Salmonella* spp.) or parasitic (*e.g. Giardia* spp.) agents.

Overall, this study revealed that the CPV strains identified through VP2 gene region sequence analysis belonged to the CPV-2b (three isolates) and CPV-2c (one isolate) variants. These results indicate the co-circulation of CPV-2b and CPV-2c strains in Türkiye. Canine parvovirus type 2c has become widespread in many countries in recent years and has been associated with more severe clinical forms in some studies (3, 10). The presence of the CPV-2b strain indicates that this classic strain is also still circulating. Various molecular epidemiological studies conducted in Türkiye have shown that the CPV-2c strain is circulating in the field (24, 25). These findings are consistent with the presence of CPV-2c detected in the current study. Currently, the majority of commercially available CPV vaccines licensed for use in Türkiye are based on the CPV-2 or CPV-2b strain, and none directly include the CPV-2c strain (1). While various studies have reported that these vaccines can provide partial cross-protection against CPV-2c, it has been noted that the level of protection may be reduced because of antigenic differences (2, 8). This situation, combined with the circulation of CPV-2c strains in the field, highlights the need to question the efficacy of current vaccines and to develop new-generation vaccines containing CPV-2c in the future. In the comparison of the Turkish CPV strains, TR-16, TR-19, and TR-32 were found to be highly similar to each other. Pairwise sequence comparison analysis demonstrated 100% nucleotide similarity (distance = 0.00) across the analysed genomic region (Fig. 3). Amino acid residues 297, 300, 305 and 323 in the VP2 protein have been identified as regions playing an important role in the host range. In this study, it was determined that these residues are conserved. In contrast, only residue 426 showed notable differences in antigenic structure, enabling its use in distinguishing between CPV-2a, 2b and 2c variants (24); it was determined that this residue had changed from aspartic acid to glutamic acid, and this change was found to be consistent with 2b and 2c based on sequence analysis. Additionally, it was found that the amino acid at position 370 of the VP2 protein, which was glutamine (Q), had changed to arginine, forming the 2c subtype (14) (Fig. 4).

In BLAST analyses of PCR products obtained from samples of dogs infected with CDV, it was determined that the two CDV sequences obtained from the study (TR-18/KNY/CDV and TR-5/KNY/CDV) showed 99% similarity to each other and were closely related to European strains. Data obtained from CDV phylogenetic analysis suggest that Turkish strains may belong to the European genetic lineage (Figs 5 and 6). The high phylogenetic similarity of these strains in Türkiye to strains commonly reported in domestic or wild mammals in Europe (4) is noteworthy in terms of regional spread, viral evolution or possible introductions. Therefore, continuous molecular-level monitoring of CDV genetic diversity and circulating strains in Türkiye is of great importance for disease control.

Analyses by BLAST of PCR products obtained from samples from CHV-infected dogs revealed that the CHV isolates obtained in this study were 97–99% identical to particular strains reported worldwide. The phylogenetic analysis of CHV strains indicated that at least two distinct CHV lineages with different genetic origins may be circulating simultaneously in Türkiye. These data indicate that CHV strains in Türkiye exhibit high genetic diversity and that both local evolution and possible external introductions may be involved (Figs 7 and 8). These differences should be taken into account in the development of control and vaccination strategies against CHV.

Positivity for CDV was detected in four nasal swab samples and CHV positivity in five, while all blood samples were negative. In a study conducted by Kim *et al*. (18) on experimentally CDV-infected dogs, it was noted that nasal and conjunctival swab samples showed a high rate of positivity in the early stages of infection (3–14 days), whereas viral RNA was detected later and for a shorter period in leukocyte (peripheral blood cell) samples. Ledbetter *et al*. (20) demonstrated that positivity was more pronounced in nasal swabs in CHV infections, while viraemia was limited in peripheral blood. These examples are consistent with the positivity observed in nasal swabs and the negativity in blood samples in the current study, supporting the notion that CDV and CHV undergo localised replication in mucosal tissues while the viraemia phase is limited.

The sensitivity of diagnostic methods for CDV infections varies by sample type; therefore sample choice is of great importance for accurate and early diagnosis. In this context, the finding that CDV positivity in nasal swab samples was significantly higher than in rectal samples in a study conducted in north-eastern Türkiye (7) is consistent with the data obtained in this study. One possible reason for this is that CDV primarily begins to replicate in the respiratory tract mucosa of infected animals. Therefore, the viral load is higher in the nasal region during the early stages of infection. Additionally, nasal swabs generally contain more cellular material, making them more suitable samples for detecting viral RNA in molecular diagnostic tests. On the other hand, positivity in rectal samples generally increases in the later stages of infection and appears after the spread of infection to the gastrointestinal system. In the present study, it was determined that age and sex did not make a statistically significant difference in the diagnosis of CDV and CHV-1 infections. This finding is consistent with that of a study in Spain (28) reporting that these variables did not affect prevalence in CDV infection, and also consistent with a PCR-based study in Iran (21) showing that these factors are similarly ineffective upon CHV infection predisposition. This suggests that age and sex are not decisive risk factors in the infection dynamics of either virus, and that other environmental and immune-related factors are likely to be more influential in the spread of infection. Therefore, in epidemiological studies, it is important to evaluate other factors that increase infection risk.

Among the results obtained, co-infection of CDV and CHV-1 was detected in one sample. Considering that there are only a limited number of reports of CDV co-infections with CHV-1 in the literature, this finding is noteworthy. Because of its immunosuppressive effect, CDV frequently predisposes dogs to co-infections with other viral, bacterial and parasitic agents. To date, in the viral category of these other agents, CPV, CAdV and various other respiratory viruses have been reported (16, 27). These co-infections exacerbate the clinical course, weaken the immune response and increase disease mortality. For example, in co-infections with CPV, gastrointestinal symptoms are more severe, while co-infections with CAdV increase the severity of respiratory system diseases (9, 33). While examples of co-infections with other pathogens are common in the literature, CDV co-infections specific to CHV-1 are limited in number; Headley *et al*. (15) reported the simultaneous detection of CHV-1, CPV, and CAdV-2 in the same dog co-infected with CDV. In that study, immunosuppression was inferred based on the presence of canine distemper virus infection and associated pathological findings, which are known to compromise host immune function. These findings indicate that viral co-infections may exhibit pathogenic synergy in immunosuppressed hosts. The immunosuppressive effects of CDV – particularly the elimination of cells producing the signal-transducing lymphocyte-activation molecule – are thought to facilitate the reactivation of opportunistic pathogens such as CHV, which can persist in a latent state.

## Conclusion

This study identified CDV and CHV co-infections in symptomatic dogs, suggesting that viral co-infections may occur naturally in this population. As immune parameters were not assessed, it cannot be determined whether CHV represented a latent or active infection. The detection of CHV in nasal swabs but not in blood samples may indicate transient viral shedding rather than systemic infection, and the clinical significance of this finding remains unclear. Nevertheless, these observations highlight the need to consider the possible presence of viral co-infections during the diagnostic process in dogs presenting compatible clinical signs. Additionally, by identifying major viral infections in the dog population in Türkiye at the molecular level, this study contributes to the understanding of local strains within the context of global genetic diversity and their phylogenetic relationships.
